# Non-Compressive, Disabling, Cervical Radiculopathy and Neck Pain: Cave Osteoid Osteoma

**DOI:** 10.7759/cureus.15209

**Published:** 2021-05-24

**Authors:** Andrea Brunori, Alberto Delitala

**Affiliations:** 1 Neurosurgery, San Camillo Hospital, Rome, ITA; 2 Neurosurgery, San Carlo di Nancy Hospital, Rome, ITA

**Keywords:** general neurosurgery, primary spine tumour, bone tumors, neuro mri, spinal pain, ortho surgery

## Abstract

Cervical radiculopathy is a common clinical condition with an annual incidence of 85/10,000. Refractory cases with positive disco-vertebral imaging findings are routinely referred to the Neurosurgeon for evaluation and treatment. In the absence of a clearcut compressive etiology, other rarer but surgically curable causes must be considered before recommending conservative management. We discuss the case of an otherwise active, healthy patient with an invalidating, refractory, relapsing nuchal pain and cervical radiculopathy. Only careful and state-of-the-art neuroimaging led to the correct diagnosis: an osteoid osteoma of the right C6 lamina was diagnosed and microsurgically resected allowing complete recovery and cure. The clinical features of these rare tumors in this unusual location are reviewed. The case is relevant for multifold reasons: it draws attention to rare conditions which can mimic radicular compression; emphasizes the need for a careful evaluation and appreciation of specific clinical symptoms and signs associated with non-compressive radiculopathies; prompts planning of a state of the art imaging workup, in order to rule out such an elusive tumor. All these measures minimize the risk of overlooking the present and other rare pathologies, sparing patients a long path of time-consuming, frustrating and cost-ineffective studies and treatment modalities.

## Introduction

Cervical radiculopathy is a relatively common (1,79-3,5/1000/years) [1-3] neurological disorder resulting from nerve root dysfunction, often due to mechanical compression. In the absence of myelopathy or significant radicular deficits, patients are treated conservatively for at least six weeks, 75%-90% of patients achieving symptomatic improvement with non-operative care [4]. Compression by disk and/or spondylotic foraminal stenosis is by far the most common cause, other intra and extraspinal conditions being less commonly involved. Among them osteoid osteoma which accounts for 10% of all benign spinal bone tumors and only 1% of all spinal tumors [5]. Cervical location accounts for only 4%-18% of all segments, the lumbar being the most affected (38%- 59%) [6,7]. Disproportion between its typically small size and magnitude of symptoms is due to extensive tumor-triggered inflammatory reaction involving surrounding neural structures, rather than direct compression. Accurate clinical history and examination along with appropriate and up-to-date neuroimaging are key to the correct diagnosis, which is frequently delayed.

## Case presentation

**Figure 1:**
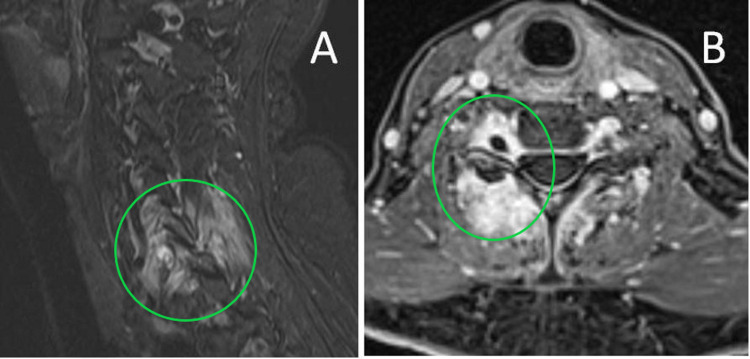
MRI study. Sagittal STIR (A) and T1-weighted Gd enhanced axial Dixon (B). Inflammatory changes in and around the spinal canal are clearly highlighted.

A 62 years old otherwise healthy woman complained of a four-month history of progressive nuchal pain radiating to the right upper limb. She also reported tingling and numbness in the C6-C7 right dermatomes. The pain became excruciating at night, often waking up the patient and causing reactive depressive changes in her mood. Although significantly relieved by NSAIDs, prolonged intake was limited by reflux esophagitis causing hiccups which further interfered with night rest. Besides tenderness in the palpation of the right paraspinal nuchal muscles, pain evoked by flexion/extension and lateral neck bent, no neurological signs were observed. MRI highlighted vast signal alteration (hypo in T1 and hyper in T2 and STIR images) involving the paraspinal tissues mainly on the right side at C5-C7, extending epidurally and along the corresponding foramina. In the same location, soft tissues strongly enhanced, consistent with increased vascular permeability. No reduction of spinal canal or foramina by disk, osteophytes or masses was detected. An infectious spinal process was suggested but ruled out based on the absence of fever and laboratory signs. Following six weeks of observation and symptomatic medical therapy, the patient underwent further MRI study including T1 pre- and post-contrast fat saturation sequences (STIR, Dixon): previous findings were better highlighted and as well as changes in bone signal in the C6 and C7 spinous processes (low in T1 and high in STIR) and C6 neural arch (Figure 1A, B).

**Figure 2:**
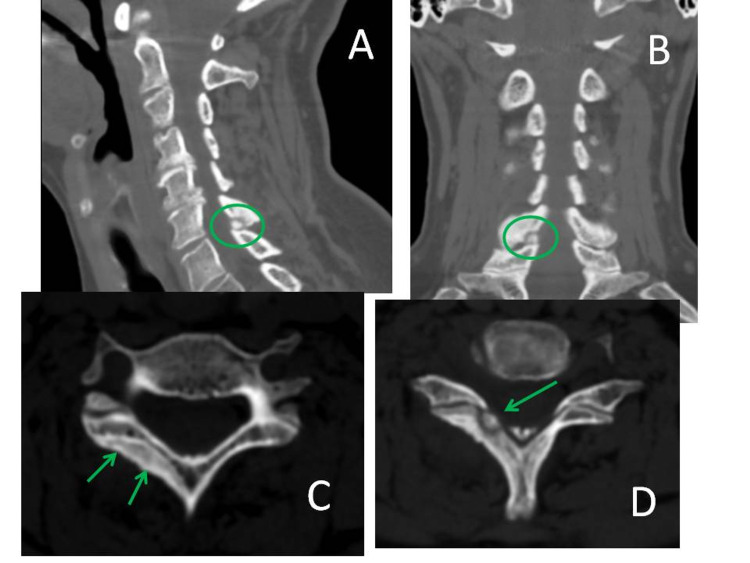
High-resolution multiplanar CT study sagittal (A), coronal (B), axial (C, D) show sclerotic thickening of the affected lamina along with the typical "target" appearance of the tumor.

The latter appeared swollen at the right hemilamina which harboured a focal heterogeneous tumor: at high-resolution multiplanar CT, the nidus showed the hallmark “target” appearance of an osteoid osteoma (Figure 2 A, B, C, D).

**Figure 3:**
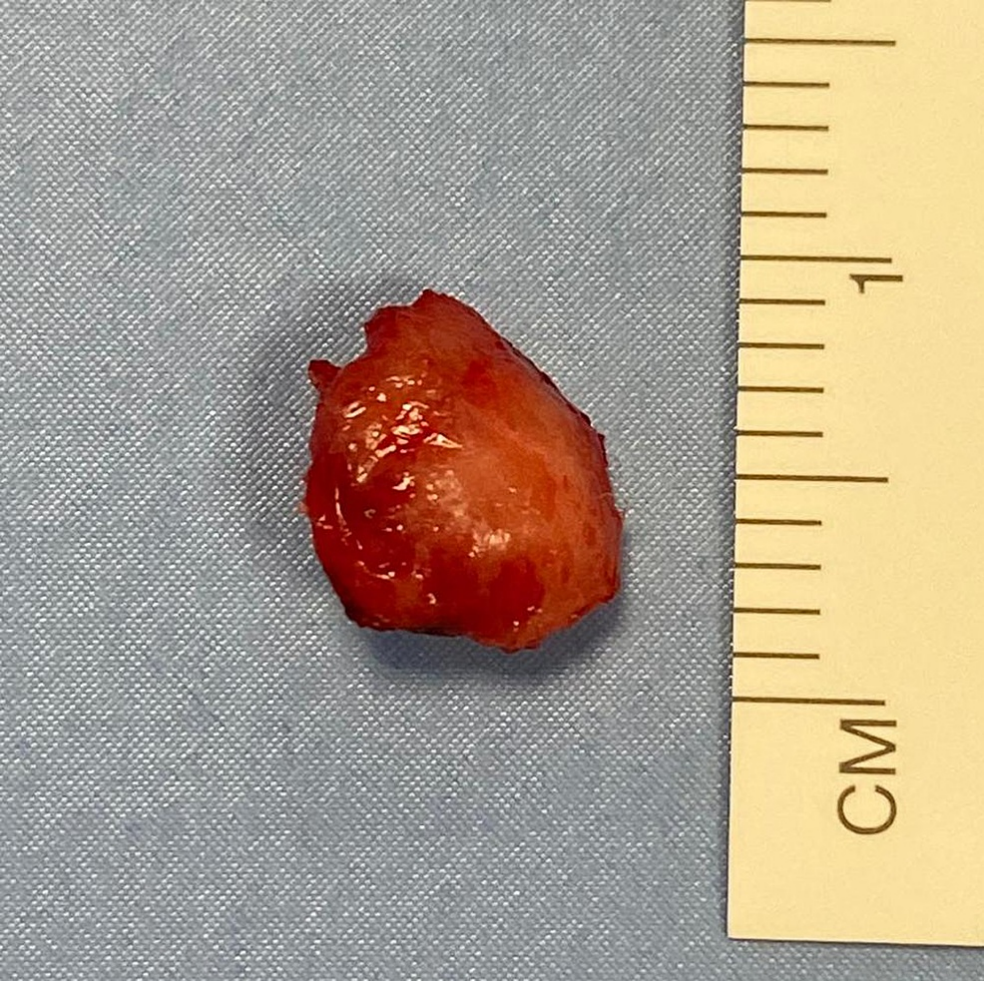
Surgical specimen.

The patient was finally referred to us and microsurgical removal adviced. A right C6 microsurgical hemilaminectomy by conventional posterior approach was performed achieving en bloc resection of the osteoid osteoma (Figure 3).

**Figure 4:**
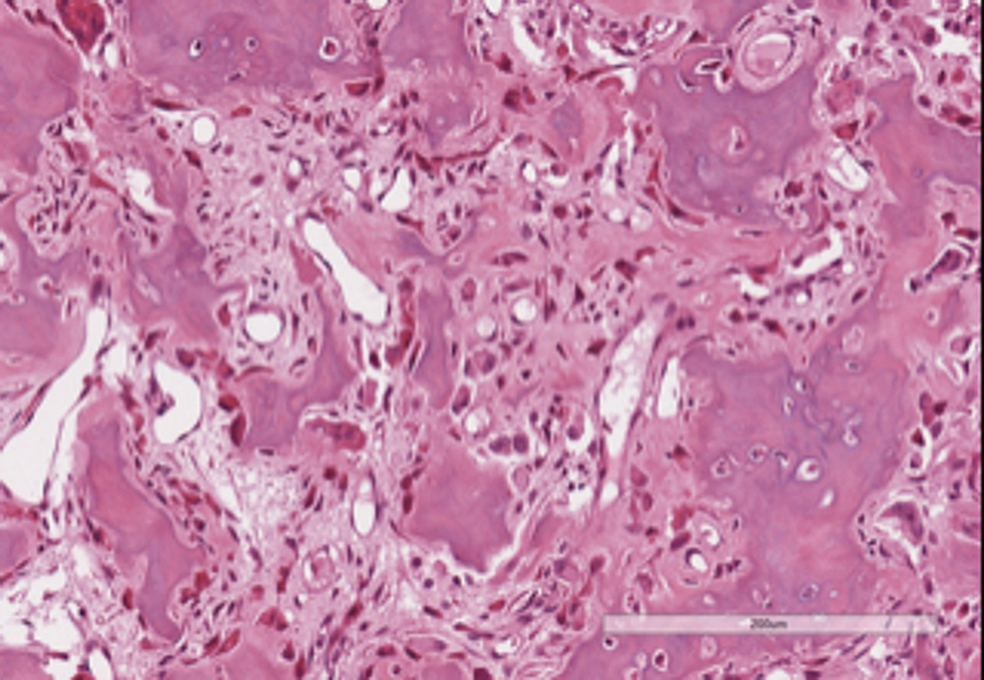
Pathological findings (HH x20) Bony spikes line by a single layer of osteoblasts and interposed richly vascular connective tissue.

The postoperative course was uneventful and the patient was discharged home on 1st postoperative day. Histology confirmed the clinical diagnosis showing bony spikes covered by osteoblasts arranged in single layer with interposed richly vascularized connective tissue (Figure 4).

At six-month follow-up, the patient has recovered completely and remains pain free without medications.

## Discussion

Cervical spondylotic radiculopathy (CSR) is one of the more frequent conditions related to dysfunction of cervical spinal nerve root. The annual incidence varies from 1,79 to 3,5/1,000 according to the most recent epidemiological studies [2,3]. Age groups above 40 years, female sex and white race are significant risk factors for this condition. Although compression by disk and bony spurs are overwhelmingly responsible, other rarer causes should not be overlooked [4,7].

Osteoid osteomas (OOs) are small (invariably less than 1.5 cm), benign bone tumors that rarely arise in the spine with a predilection for the neural arch (75%) [5]. Levelwise, half of spinal cases involve the lumbar spine while only 4%-18% arise in cervical segments [6,7]. A slight prevalence of males (1:1 to 2.5:1) and young age (<30 years) has been reported [8]. The neoplasm typically consists of a highly vascularized nidus (bony trabeculae with osteoblast lining, free unmyelinated nerve endings) surrounded by a rim of sclerotic bone [5,9]. Malignant transformation has been reported and corroborates indication to resection [10], Intense synthesis and secretion of prostaglandins (PGE2) [9] triggers progressive inflammatory reactions in the surrounding tissues: this biochemically mediated mechanism, underlies the flourishing clinical picture whereas compression of neural structures by the lesion itself is exceptional. Neck pain, local tenderness , stiffness of paraspinal muscles and antalgic scoliosis are the most common symptoms [8]. Pain typically becomes more severe at night waking up 29% of patients. Response to NSAIDs is invariably observed, confirming the inflammatory PGE-mediated pathogenetic theory. These hallmark findings are confirmed by our own observation.

While axial pain is the rule, radicular involvement with clearcut radiculopathy in OOs are much rarer; in most such cases compression by the tumor is advocated, while to our knowledge only two non-compressive cases have been described to date: the Authors documented intense periradicular infiltration by lymphocytes and plasma cell at histology, in a T 11 OO [11]. Although lacking histological confirmation a similar mechanism can be advocated in our patient based on prominent MRI inflammatory findings.

The diagnosis of spine OOs is challenging and often delayed due to a specific symptoms and the small size of the lesion which can be easily missed not only at plain X-rays in classic projections but also at CT if appropriate slice thickness, orientation or multiplanar reconstructions are not carried out. While radionuclide scanning has been indicated as a valuable localizing tool [5,7,11], MRI is considered the state-of-the-art investigation since the redundant inflammatory changes in soft tissues and bone oedema associated to OOs are best highlighted. The nidus of an OO is intermediate to low signal intensity on T1-w images while shows variable signal on T2-weighted sequences depending upon the amount of mineralized matrix within the nidus. The non-mineralized matrix is typically high signal intensity on T2-weighted sequences [12]. The nidus may present a "target‟ appearance on T2-weighted images with low signal intensity centrally surrounded by increased signal. This signature feature is however best depicted at CT: a central sclerotic dot within a round, a transparent nidus, limited by a rim of sclerotic reactive bone, as in our case. The vascularity of the nidus is especially appreciated in dynamic contrast images: intense enhancement begins in the arterial phase (30 seconds post-injection) while washout causes camouflage of the signal by bone marrow during the venous phase (90 seconds). This typical pattern has been shown to be present in 83%-100% of OOs [12].

Our case however demonstrates that good depiction of reactive changes in the vicinity of the tumor by use of adequate MRI sequences is the key to correct diagnosis. In our experience, fat saturation techniques have been proven especially valuable: among them, Dixon technique allows a more uniform and less affected by artifacts suppression of fat signal than many other techniques [13]. The advantages of Dixon technique are multifold: they can be combined with a variety of sequence types (spin echo, gradient echo, steady-state free precession sequences) and with a variety of weightings (T1, T2 and proton density), providing multiple images from a single acquisition, with and without fat suppression. This technique is superior to SPAIR for the quality of fat suppression and for the delinea­tion of lumbar spine lesions [14]. Some Authors have proposed that a single sagittal T2-weighted Dixon sequence may replace the recommended combination of T1, T2, and fat-suppressed T2 weighted sequences in the standard diagnostic protocol of non-specific low back pain and lumbar radiculopathy [15]. In our case, both STIR and especially Dixon sequences highlighted an area of edema and increased permeability extending from the epidural space and C6-C7 right foramen to the lamina and paraspinal muscles predominantly on the right with wide bilateral extension. These findings guided the CT scan on the correct area, expediting the correct diagnosis.

The treatment of choice for OOs is surgical resection. Depending mainly on the location and accessibility of the lesion, open surgery and CT-guided radiofrequency ablation (RFA) have been advocated. Pipola et al. [7] compared a large cohort of spinal OOs treated by open surgery (58 pat) or RFA (80 pat) the average follow-up being 57.45 and 62.75 months, respectively. Recurrence rate was significantly higher in RFA group (12.5 vs 1.7%), with regrowth occurring at a mean of 11.6 months from surgery. Lesions in close proximity of critical neurovascular structures should be excluded from RFA treatment due to the risk of uncontrolled thermal injury. In surgical cases, they emphasized the need to achieve resection margins in normal tissue and the use of high-speed drill. Only 12% of patients in the surgery group underwent instrumented fixation as sporadically reported in other smaller series and reports: since most vertebral lesions (75%) arise in the neural arch, en bloc removal is often feasible without compromise of stability as in our case. A minimally invasive transmuscular approach, as recently described by Jannelli et al. [16], could offer patients an even smoother postoperative course, providing the same radicality.

## Conclusions

Cervical radiculopathy in the absence of disco-vertebral compression should be carefully evaluated for other potentially surgical causes, OOs among them. High-quality MRI studies are crucial in the imaging workup, since adequate sequences, namely fat suppression techniques (STIR, Dixon) can best define the inflammatory changes in the surrounding tissues, triggered by the secretion of PGE from the tumor nidus. The nidus though can be easily missed due to the small size and variable signal features. High-resolution CT scan with multiplanar reconstructions targeted on MRI indications is therefore a compulsory adjunct in the OO presurgical workup. The PGE biochemical storm involving muscles, ligaments, bone and neural structures within the spinal canal underlies the characteristic clinical syndrome (nocturnal neck pain, tenderness of nuchal muscles, antalgic scoliosis) and justifies excellent, though transient response to NSAIDs. Radiculopathy is a less common feature in OOs and has been seldom reported. Open surgery is the gold standard of treatment: resection in healthy bone margins provides negligible recurrence rates and is preferable to RFA, in the vast majority of patients. Additional instrumented surgery is seldom needed.
